# American Medical Association

**Published:** 1869-05

**Authors:** 


### Miscellaneous.

From the New Orleans Republican.

### American Medical Association.

First Day.—The first meeting of the American Medical Association ever held in New Orleans assembled yesterday morning in the room in the Mechanics' Institute, once used as the Senate Chamber of the Louisiana General Assembly.

It is an annual session, and was called to order by the President, Dr. William O. Baldwin of Alabama, who invited to the platform the vice-presidents and ex-presidents of the Association, and then, noticing his presence, invited "the distinguished Dr. Stone" to the stand. Dr. Lopez, one of the former vice-presidents of the Association, was also invited to the platform.

Prayer by Rev. Mr. Gallaher of Trinity Church.

The report of the Committee of Arrangements was made by the Chairman, Dr. T. G. Richardson of this city, which was an address of welcome to the delegates from the various States who meet here as a National Medical Association, rather than a dry, uninteresting detail of how the arrangements were made.

If we remember correctly, a resolution was introduced some time since into one of the meetings of the Medical Association of New Orleans, by Dr. George W. Dirmeyer, inviting the American Medical Association to meet here as they have done. The Committee of Arrangements consisted of Dr. T. G. Richardson, chairman; Dr. S. M. Bemiss, Dr. C. Beard, Dr. S. Choppin, and Dr. W. S. Mitchell of New Orleans, and they have performed their duties most creditably.

After the report of the committee was read, Dr. Richardson made a verbal report that the committee saw no reason for a change in the hour of meeting, which was ten o'clock in the morning, and to adjourn at two o'clock. He gave notice also of the places of meeting of three sections or committees, and then proposed the nomination of several medical men as members of the Association by invitation. Among the names was that of Dr. G. W. Dirmeyer.

*The Annual Address.*

The President, Dr. Baldwin, then read the Annual Address, of which we give an abstract:

Gentlemen of the American Medical Association:

I congratulate you on the return of an occasion which permits us to renew that fraternity of intellect no less than that sympathy of feeling by which our life and vocation as physicians are beautified and ennobled. Of no profession are the inspired words more true than ours, that we are "members one of another." The ideal of our profession is that of complete and thorough oneness. What is scientific truth for one is scientific truth for all. We have a common estate in the facts, aims and purposes that belong to the science of medicine; and hence we do a wise work when we acknowledge the exalted unity of the medical profession by this annual assemblage.

The nature of this occasion reconciles me, in some degree, to the task which I now have to perform. When I remember that the position I occupy was first filled by the distinguished Chapman, and that the succeeding anniversaries have been presided over by men whose genius had shed not only light, but lustre, on the annals of our profession, I feel that nothing but the inspiration which breathes through affections, kindled into life by this Association, could sustain me under the sense of incompetency for the duties to which your kindness has called me. Relying on the same spirit which prompted you to confer on me the highest distinction within the gift of the medical profession of America; and hoping that my deficiencies may be forgotten in the interest and magnitude of the subjects awaiting your deliberation, I proceed to discharge the duty which the custom of my predecessors has imposed upon your presiding officer.

The spirit of a profession is the true sign of its character, as it is the measure of that respect with which its talents and services are regarded. Manly sentiment, springing from broad and genial sympathies, is the soul of every profession, and if it is wanting, no skill, not even usefulness, can prevent it from sure and speedy degradation. The first and last requisite of professional life is not power of intellect, however valuable that may be, nor those acquisitions of knowledge that enrich our thoughts, but that other and finer quality of generous manhood which, as a subtle and pervading essence, enters with its healthy vigor and animating impulse into all its connections. Of our profession this is eminently true; and, on this account, I rejoice that the records of this Association give no evidence of sectional unkindness and prejudice even during the period of our bloody war.

To me, gentlemen, this occasion is one of solemnity and significance. Standing here in the great commercial metropolis of the South, I find myself surrounded by men representing nearly every section of a country so lately arrayed in hostile strife. At a time when every other organization has been shaken to its center by the passions of deadliest hate; at a time when the most matured conservatism has been over-mastered by the vindictive fury which has swayed the popular mind; at a time when even instinct has been treacherous to its ends; you have been drawn hither from homes far distant, over highways full of painful historic incidents, through territories watered by the blood and tears of a sorrowing nation; and you have assembled here as brothers and friends, to unite your offerings to a common science. The mournful witnesses of this terrific struggle have confronted your eyes; the shadowy phantoms still linger on the stage where these tragedies have been performed; the air we breathe has not yet lost its echoing groans of dying heroism nor the pathetic anguish of sorrowing relatives. Amid these circumstances, so sundering to the most sacred companionships of life, you have met in the spirit of Him who is this world's greatest and best Healer—that Divine One, who, opening and continuing his ministry of service by curing all manner of diseases, finished its majestic self-denials in the reconciliations of the cross.

Eight years ago we were separated by civil war. That war engendered the bitterest feeling in every other national organization, whether scientific, political, or Christian; but the members of this Association, without words of crimination or reproach for one another, assumed the respective places assigned them by the obligations of citizenship. Through the long and bloody contest which ensued, this Association, in its resources, honor and renown, was in the keeping of our Northern brethren; and during those memorable years, when the sense of bitter wrong and burning hate filled all hearts, and when friendships and affections, born of the hallowed ties of friendship and consanguinity, sent their messages—once of love and tenderness—at the point of the bayonet or through the cannon's mouth, what were the feelings which moved this Association? At the first meeting, two years after the war began, they indulged only in expressions of profound regret that "the brethren who once knelt at the same holy altar, and drank with them at the same pure fountain,"* had been separated from them by civil war, endangering thereby the claims of the Association "to an unselfish nationality, and robbing it of the presence and counsel of many of its warmest adherents,"† while praying at the following meeting that the period would soon come when we should again be "one in our political, professional and social relations."‡

The same humane and catholic spirit continued during the war to mark the conduct of the members of this Association. Each of the divided sections met the tasks required by its respective position. But wherever found, whether sharing the hardships of the campaign or discharging the duties of private practice, they comprehended the essential difference between what might prove on the one hand a transitory evil, and what on the other hand they knew would be a lasting good. Accordingly they remained the constant representatives of a noble brotherhood. If they did not sink the patriot in the physician, they did not sink the physician in the patriot. The imperative instincts of each character, true to its trusts and faithful to its requirements, acted for themselves and in the direction of their own ends. Amid the shouts of battle and the shock of arms they raised themselves to the height and grandeur of their calling, and thus stood far above the embittered prejudices that encircled all other classes of men. So far from allowing the fugitive passions of the times to betray them from their professional allegiance, they vindicated their sagacity no less than their manliness by looking to the future—by contemplating results not the less certain because remote—by regarding with thoughts chastened and subdued that state of man in which the interests of life and death meet together—and by considering as paramount to all selfish motives the claims of that science with whose undisclosed mysteries they must yet wrestle for the well-being of mankind. Above all, they looked to the transcendant

* Transactions, 1863. † Transactions, 1863. ‡ Transactions, 1864.

value of a virtue, which should contrast in broad masses of light, its purity and power with the corruptions and frailties of the hour, which should by reason of its disinterestedness diffuse itself through the affections of nations and reach in the large outgoings of its sympathy the hearts of generations yet unborn.

When at last this dispensation of carnage ended, and while as yet the war-path was crimsoned with the blood or whitened with the unburied bones of our brethren, this Association again met. Like the surges of the sea, dark, tumultuous, raging, though the storm has passed from the sky and fled beyond the horizon, the meaner instincts of hatred, revenge and persecution still swayed the multitude. The mob of fanatical intellect unappeased, and the mob of popular passions thirsting for new strife, joined their hands to prolong the wretched alienation. The avenging angel had lifted his brooding wings from the landscape and cried, "It is enough;" but now other vials of wrath seemed to be about poured forth on a land hopeless because helpless. You then met to pour oil on the unquiet waters. Here was scope for a statesmanship, aye, for a generalship grander than any which the war had developed. Here was the best of opportunities to inaugurate a new epoch of fraternal sympathy. Nor were you unmindful of its solemn behests. True to your past professions of regret over our separation, you saw the vacant seats in this Association of your Southern brethren, and, actuated by the higher instincts of manhood, and scorning the base ambition to degrade a fallen antagonist, whom the saddest experience had taught the bitterest lessons of life, you set the nation an example of dignity, moderation and virtue, to which no other organization in the land has yet had the wisdom or the sensibility to rise.

Within a few weeks after the cessation of hostilities, this Association held its regular annual meeting in the city of Boston, and there renewed, with manly sympathy, its former expressions of kindness, inviting us to come again and be their brethren. I quote their own language on that occasion when I say: "The unhappy feud which for years has divided the nation has ceased, and peace has come, we trust forever; so we hope soon again to meet our members and delegates from the South on the platform of fraternity, and to this end we extend to them a cordial welcome."* At a subsequent meeting you repeated this sentiment in the following language: "We would fain meet again those from whom we have been separated, draw the mantle of forgetfulness over the past, renew to them the expression of regard, and with them dedicate the hour and the occasion to the sacred cause of learning, friendship and truth."†

And when at the last meeting we met our Northern brethren, how were we received? They met us as equals in the past and equals in the present, saying in effect if not in words: "If quarrel we ever had, it is over; we have no explanations to offer, no apologies to demand; we know that we have done our duty; we feel that you have done no more, and that you would have been unworthy your noble vocation had you done less; we have guarded faithfully the institution so long left in our charge, in which we now claim but an equal interest with you; with the incense we have burned in its sacred fane we have not permitted the poisonous spirit of party to mingle, and we now invite you to go with us to the smiling and peaceful fields of that science whose interests it shall be our common work to foster and advance; here we will walk with you to the stern realities and moveless grandeur of labor and thought, and find in their quiet paths a relief from the gloom of the past; here we will divide with you the toils and share with you the rewards of labor, the honors of success."

Against the insolence of the day; against its unreasonable pride, its overweening vanity and its shameless scorns, your conduct bore a moral protest, which, while acting directly on our profession, has had no small agency in producing those indications of a return to reciprocal sentiments of confidence and respect in which all the good men of the country rejoice. The mythical war between the Athenians and Amazons led, in the midst of arms, to the most intimate friendship between their leaders. When Pirithous and Theseus finally met on the plains of Marathon, after many a hard fought battle, the former, regarding himself and army as captives, said to the latter: "Be judge thyself; what satisfaction dost thou

* Resolution introduced by Dr. J. R. Van Blesk of New York, and adopted. Transactions for 1865, vol. 16, p. 57.

† Address of Committee of Arrangements, through their Chairman, Dr. C. C. Coxe. Transactions for 1866, vol. 17, p. 10.

require?" The noble Athenian replied: "Thy friendship;" and they swore inviolable fidelity, and were ever after true brothers in arms. Alas, that the nineteenth century has so often to recur to classical heathenism to find its illustrations of genuine magnanimity!

Looking at these facts, am I not warranted in asking if any organization has emerged from our late convulsions with so much dignity? Has it not come forth from the sharp ordeal with those graceful virtues that belong to our higher nature? The world may have its conventional rules of intercourse between man and man—its creed of moral philosophy—its code of honor—its accredited formula of behavior, while it lavishes its praise on the charms of human brotherhood; but it has been left to the American Medical Association to teach practically the intellects of the land one of the most ennobling lessons in the dignity, beauty and glory of refined and civilized life—a lesson that not only hallows the spirit of our professional character, but instructs the physician in those spiritual sentiments which lead to the highest virtues, among which are reckoned Charity and Forgiveness. Of the one, we are told, that the Archangel, who never knew the feeling of hatred, has reason to envy the man who subdues it; while of the other, it is said, that when we practice forgiveness to the man who has pierced our heart, he stands to us in the relation of the sea-worm that perforates the shell of the mussel which straightway closes the wound with a pearl.

No apology, gentlemen, is necessary for dwelling so long on the moral spirit of this Association. If I had not believed that a moral sentiment underlies all profound thought, all true research, all genuine wisdom; that it is the strength of civilization, the security against those court forms of heathenishness and brutality that lurk under the imposing hypocrisies of outward splendor, and the ulterior end for which nations and mankind exist; if I had not been assured that our profession rests on this basis, and can rest on no other, I should not have devoted so much time to this subject. Turning from these reflections, so naturally suggested by the circumstances of our present meeting, I am reminded that other points of great practical significance claim our attention.

Dr. Baldwin then proceeded to examine the subject of medical education in its practical bearings. Basing his remarks upon the fact that this subject presented itself under new aspects, Dr. Baldwin offered some most valuable and well considered suggestions, which we regret that want of space forbids us from giving. Throughout this branch of the address, which was listened to with profound attention, the President insisted that it was the duty of the Medical Association, originally organized for specific objects, the chief of which was the elevation of medical education, to vindicate its claims to that high mission, by holding to a stricter accountability both those who bestow and those who receive diplomas in medicine.

After the address was concluded, Dr. Eve of Tennessee, moved an adjournment for five minutes. This motion prevailed.

At one o'clock the session was resumed. Letters were read from absent members, explaining the reasons why they were unable to be present.

Reports of committees were called for. Dr. Lemuel J. Deal of Pennsylvania, chairman, reported on the cultivation of the cinchona tree. The report was received and referred.

On alcohol and its relations to medicine, a report from Dr. John Bell of Pennsylvania, chairman, was referred.

Report on the cryptogamic origin of disease, with special reference to recent microscopic investigations on that subject, Dr. Edward Curtis, U. S. Army, chairman, was referred to Committee on Epidemics.

Report on prophylactics in zymotic diseases, Dr. Nelson L. North, chairman, was referred to the Committee on Epidemics.

Report on nurse training institutions, Dr. Samuel D. Gross of Pennsylvania, chairman, was referred to Committee on Practice of Medicine.

Report of commissioners to aid in trials involving scientific testimony, Dr. John Ordronaux of New York, chairman, was referred to the Committee on Medical Jurisprudence.

Report on annual medical register, Dr. John H. Packard of Pennsylvania, chairman, that want of funds rendered the committee unable to accomplish anything.

Dr. Richardson of Louisiana, thought the plan of publication of a register was practicable, and that through the instrumentality of the Postmaster General a report could be obtained from postmasters of the names of all practicing physicians in the United States.

Dr. Mussey of Ohio, considered this impracticable, and offered a resolution that each State Medical Society be requested to prepare an annual register of all the regular practitioners in their respective States, giving the names of the college in which they have graduated and date of graduation.

Report on devising a plan for the relief of widows and orphans of medical men, Dr. John H. Griscom of New York, chairman, recommended a general plan of mutual aid association. Read and referred.

Report on specialties in medicine, and the propriety of specialists advertising, Dr. E. Lloyd Howard of Maryland, chairman, was submitted, and made the special order at twelve o'clock to-day.

Report on library of American medical works, Dr. J. M. Toner of District of Columbia, chairman, was submitted, and made the special order for one o'clock to-day.

Report on the best method of treatment for the different forms of cleft palate, Dr. J. B. Whitehead of New York, chairman, was referred to the Committee on Surgery.

Report on medical ethics, Dr. D. Francis Condie of Pennsylvania, chairman, was accepted.

No report on American medical necrology, but a recommendation in reference to continuing the committee was adopted.

Various essays were presented, and appropriately referred.

On the climatology and epidemics of the various States, Dr. T. J. Heard of Texas, made a report, which was referred to the Committee on Climatology and Epidemics.

The Committee on Epidemics was authorized to fill vacancies in any of the States.

The report of committee of last year on amendment of the organization of the Association was made the special order for this morning at ten o'clock.

Communication from the Medical Association of Cincinnati, recommending the appointment of a committee to draft a law regulating the practice of medicine in the different States.

Resolutions by Dr. Charles H. Lee of New York, providing for medical examiners to examine all who desire to practice medicine by each State Association to examine all who desire to practice medicine in the State.

Communication from the Medical Association of Waco, Texas, expressing sympathy with the noble objects of the American Medical Association, and submitting certain recommendations.

These several resolutions and communications were referred to a committee of five, to whom also other similar papers are to be referred, to be appointed by the President.

Announcement of places of meeting of committees were made, and the Association adjourned till nine o'clock, May 5th.

SECOND DAY.—The attendance is much larger than yesterday. A number of arrivals has increased the *personnel* of the Association to near three hundred. The hall is well filled, and the same dignified deportment which marked the deliberations of the members on the opening day is displayed to-day. Much of the facility with which the deliberations were conducted yesterday, and the promptness with which action was taken upon the various resolutions, was, undoubtedly, due to the tact and practical administrative knowledge displayed by President Baldwin.

At 9 A. M. Dr. W. O. Baldwin, the President, in the chair, called the meeting to order.

A paper on "Canula and the New Mode of Applying Ligatures" was submitted by Dr. P. F. Eve, (Tenn.) and was referred to the Section on Surgery.

Dr. J. M. Bush of Kentucky, offered the following resolution:

*Resolved,* That a committee of five members be appointed by the chair, to take into consideration the subjects alluded to in the President's address, and report at this meeting.

This resolution having been adopted, the President selected as members of the committee Dr. Parvin of Indiana, chairman; Dr. Toner of the District of Columbia; Dr. Pollock of Pennsylvania; Dr. Welch of Texas; Dr. Seeley of Alabama.

Dr. McPheeters of Missouri, offered a communication from the Medical Association of that State, in reference to medical education.

On motion of Dr. Toner, it was referred to the special committee on that subject.

Dr. Eve offered the minutes of the Medical Society of Tennessee, which was similarly referred.

Dr. Gaillard of Kentucky, offered the following preamble and resolutions, which were referred to the same committee:

*Whereas*, The medical teachers of America have, after a trial of twenty-two years, failed to meet satisfactorily and efficiently the requirements of the great body of the profession in regard to medical education; and

*Whereas*, The condition of the profession is yearly becoming more deplorable on account of the antagonistic and objectionable policy of medical schools, in making the amount of fees charged, rather than scientific teaching, the basis of competion; and

*Whereas*, To obtain professionally competent graduates, sound and efficient teachers are indispensably necessary; and

*Whereas*, Such teachers, to be found throughout the country, cannot be induced to leave their homes without the assurance of competent remuneration; and

*Whereas*, Such remuneration can only be obtained by adequate fees charged, unless by a system of low fees the number of students be relied upon to make up the inevitable pecuniary deficiency; and

*Whereas*, Reliance upon numbers of students for this purpose deplorably crowds the already overcrowded professional field, diminishing thereby individual income, judgment, experience, and skill, thereby compelling practitioners to resort to other avocations as a source of supplemental income; and

*Whereas*, This devotion to other pursuits destroys opportunities for study and improvement, degrading thereby the status and standard of American physicians; and

*Whereas*, The schools of New England, New York, Pennsylvania, Maryland, Virginia, South Carolina, Georgia, Missouri, Tennessee, Louisiana, Alabama and District of Columbia, now charge comparatively remunerative fees; and

*Whereas*, The *low system* of fees is charged only in a few of the Middle States, and can with advantage be made to conform to the rate of fees charged elsewhere; and

*Whereas*, It is as unethical for colleges to underbid each other pecuniarily, as it is for practitioners to do so.

*Resolved*, That hereafter no medical school in this country, other than those fully endowed, be entitled to representation in this Association, if the amount charged by such schools for a single course of regular lectures be less than one hundred and forty dollars.

*Resolved*, That all schools charging less than this sum are earnestly requested by this Association to advance their rate of fees to the amount mentioned.

The report of Dr. Lee of New York, the delegate to the Association of Superintendents of Insane Asylums, was offered and referred to the Section of Psychology.

The report of Dr. Gross of Pennsylvania, delegate to Foreign Medical Association, was presented, together with the letter to Dr. Ehrenberg, was read and referred to the Committee of Publication.

The time having arrived for consideration of the revision of plan of organization, it was, on motion, taken up.

A recess was taken to allow the selection of members of the Committee on Nominations.

On re-assembling, the Permanent Secretary announced the following as the Committee on Nominations:

New York, J. C. Smith; Delaware, H. F. Askew; Pennsylvania, A. M. Pollack; Kentucky, H. M. Skillman; Tennessee, J. B. Lindsley; Mississippi, W. Y. Gadbury; Alabama, J. Cochran; Ohio, John Townsend; Indiana, B. S. Woodworth; Illinois, T. D. Fitch; Wisconsin, H. Van Dusen; Missouri, J. S. Moore; Michigan, J. B. White; Georgia, R. D. Arnold; Louisiana, S. Logan; Texas, S. M. Welch; Minnesota, C. N. Hewitt; Arkansas, R. G. Jennings; West Virginia, W. J. Bates; Rhode Island, G. L. Collins; District of Columbia, L. W. Ritchie; U. S. Army, J. J. Woodward; U. S. Navy, F. E. Potter.

Dr. Chaille of Louisiana, submitted a proposition for a common medical nomenclature in the United States, taking as a model an official publication on the subject by the Royal College of Physicians of London, and offered the following resolutions, which were adopted:

*Resolved*, That a committee of five be appointed by the President to report as soon as practicable to the present session of this Association upon the following:

1. The propriety of adopting and using its influence to have adopted by the entire medical profession in the United States the provisional "Nomenclature of Diseases of the Royal College of Physicians."
2. On the practicability of having this nomenclature published in such manner as may render it easily and cheaply accessible to every member of the profession.
3. To recommend such other practical measures for the action of this Association as may be necessary to introduce this nomenclature into official (military, naval, etc.) and general use.

The Chair appointed the following gentlemen as the committee: Drs. Woodward, U. S. A.; Houstis, of Alabama; F. G. Smith, of Pennsylvania, and Chaille, of Louisiana.

The reports of the Committee of Publication, and the Treasurer were read, accepted and referred to the Committee of Publication.

On motion, the Committee on Nominations were permitted to retire for consultation.

The special order for 12 being the report on Specialists, it was read by the Secretary, and, on motion of Dr. Sayre, the resolutions were adopted and the report referred to Committee of Publication.

The large number of arrivals has increased the *personnel* of the Association to near four hundred.

Dr. L. P. Yandell, Jr., of Kentucky, introduced the following resolution:

*Resolved*, That private handbills addressed to the members of the medical profession, or advertisements in newspapers, calling the attention of professional brethren to themselves as specialists, be declared in violation of article —— of section —— of the Code of Ethics of the American Medical Association.

Dr. S. N. Davis of Illinois, said it had been the practice to publish cards in medical journals for the purpose of informing the medical fraternity that the advertiser devotes himself to special diseases. These cards were not so much for the information of the public, as for the medical fraternity. He hoped that, now the question was up, it would be discussed fully.

Dr. L. P. Yandell of Kentucky: We have allowed physicians to violate the code of ethics by advertising in medical journals that they are specialists in the treatment of certain diseases. In Europe they are stricter in regard to specialists than here. There, where a physician wins a reputation in the treatment of certain diseases, his professional brethren send cases to him for treatment, but advertisement is prohibited. If we are allowed to resort to advertisements, not as a question of merit, but of money, this Association should so declare. I am sure I am right in principle, and I want to get an expression from this Association.

Dr. Sayre of New York: Let those who understand the best mode of treatment in special diseases instruct their professional brethren through the proper channels, as the honorable way of preferment, not by advertising as a matter of dollars and cents. Let us look the matter square in the face and sustain the resolution of Dr. Yandell. May my hand be paralyzed if I make any attempt to profit by advertising the knowledge I have attained in my profession.

Dr. Mussey of Ohio, moved to amend by inserting "or in medical journals."

This amendment was accepted.

Dr. Davis of Illinois: The question before us is one to settle the interpretation of the existing statute. The question is whether that rule of ethics shall be enforced prohibiting publication of cards calling attention of individuals laboring under particular diseases.

Dr. Yandell stated that he had preferred charges against a practitioner of Louisville for advertising himself as a specialist in the newspapers, and for sending handbills to physicians, but that the medical societies of that city not having sustained him he brought this subject up for the action of this Association. The question is, shall we associate with professional prostitutes and medical outlaws?

Dr. Yandell's resolution was unanimously adopted.

The Committee on Prize Essays reported as follows, their report being adopted:

They have received but two essays—one upon "The Physiological Effects and Therapeutical Uses of Atropia and its Salts;" the other upon "Quinine as a Therapeutic Agent." They agree to present both of these essays to the Association, and to recommend the award of a prize of one hundred dollars to each of them.

S. M. Bemiss, Chairman.

The Secretary broke the seals, and announced that Dr. S. S. Herrick of New Orleans, was the author of the paper on Quinine, and Dr. Robert Bartholow of Cincinnati, was the author of that on Atropia.

Dr. Booth of Mississippi, offered the following preamble and resoluion:

*Resolved*, That the proper construction of Art. 4, Sec. 1, Code of Ethics, A. M. A., having been called for, relative to consultation with irregular practitioners who are graduates of regular schools;

*Resolved*, That said Art. 4, Sec. 1, Code of Ethics, A. M. A., excludes all such practitioners from recognition by the regular profession.

This resolution was unanimously adopted.

On motion, the Association adjourned until Thursday, at 9 A. M.

(To be continued.)

---

Dr. Alexander H. Stevens, late President of the New York Academy of Medicine, died on the 30th of March last, at the advanced age of eighty years.

